# Real-Time Trajectory Prediction Method for Intelligent Connected Vehicles in Urban Intersection Scenarios

**DOI:** 10.3390/s23062950

**Published:** 2023-03-08

**Authors:** Pangwei Wang, Hongsheng Yu, Cheng Liu, Yunfeng Wang, Rongsheng Ye

**Affiliations:** 1 Beijing Key Lab of Urban Intelligent Traffic Control Technology, North China University of Technology, Beijing 100144, China; 2 Key Laboratory of Operation Safety Technology on Transport Vehicles, Research Institute of Highway Ministry of Transport, Beijing 100088, China

**Keywords:** intelligent perception, real-time trajectory prediction, multi-sensor data fusion, improved LSTM, intelligent connected vehicles

## Abstract

Intelligent connected vehicles (ICVs) have played an important role in improving the intelligence degree of transportation systems, and improving the trajectory prediction capability of ICVs is beneficial for traffic efficiency and safety. In this paper, a real-time trajectory prediction method based on vehicle-to-everything (V2X) communication is proposed for ICVs to improve the accuracy of their trajectory prediction. Firstly, this paper applies a Gaussian mixture probability hypothesis density (GM-PHD) model to construct the multidimension dataset of ICV states. Secondly, this paper adopts vehicular microscopic data with more dimensions, which is output by GM-PHD as the input of LSTM to ensure the consistency of the prediction results. Then, the signal light factor and Q-Learning algorithm were applied to improve the LSTM model, adding features in the spatial dimension to complement the temporal features used in the LSTM. When compared with the previous models, more consideration was given to the dynamic spatial environment. Finally, an intersection at Fushi Road in Shijingshan District, Beijing, was selected as the field test scenario. The final experimental results show that the GM-PHD model achieved an average error of 0.1181 m, which is a 44.05% reduction compared to the LiDAR-based model. Meanwhile, the error of the proposed model can reach 0.501 m. When compared to the social LSTM model, the prediction error was reduced by 29.43% under the average displacement error (ADE) metric. The proposed method can provide data support and an effective theoretical basis for decision systems to improve traffic safety.

## 1. Introduction

With the rapid development of 5G communication and intelligent connected vehicles (ICVs), the trajectory prediction of ICVs under a vehicle-to-everything (V2X) [1] system has become an important technology to improve the service level of ICVs [2,3,4]. Meanwhile, considering that transportation requires high levels of efficiency and safety, the accuracy and lower latency performance of the trajectory prediction methods need to be further improved [5,6,7].

The collision risk between vehicles existing in urban intersections can be reduced effectively by predicting the trajectory of ICVs [8,9]. The first step of trajectory prediction is target detection and tracking, which can provide reliable and microscopic multidimension data for the real-time prediction of ICV trajectories, and the target detection methods are mainly based on multi-sensor data fusion (MSDF) technology. As deep learning technologies are applied more and more widely within multilayer data coupling, vehicle perception technologies are also rapidly developing based on camera and light detection and ranging (LiDAR) sensors [10,11,12]. Jie et al. [13] proposed an optimal attribute fusion algorithm for target detection and tracking based on a Gaussian mixture probability hypothesis density (GM-PHD) filter, which could output stable classification information and high-accuracy positioning and tracking information from the target.

Perception methods for vehicles can reduce the rate of accidents at an intersection. Meanwhile, improvements in the performance of control algorithms also need the support of real-time and accurate prediction data [14,15]. Based on perception methods, the trajectory prediction methods gradually became the focus of research. Schreier et al. [16] used an integrated Bayesian approach with a Monte Carlo algorithm to achieve trajectory prediction for longer time domain intervals. When combined with an unscented Kalman filter (UKF) and a dynamic Bayesian network, Xie et al. [17] proposed the interactive multiple model trajectory prediction (IMMTP) methods to predict vehicle trajectories accurately in specified scenarios. In recent years, vehicle trajectory prediction methods based on neural networks with autonomous learning capabilities have gradually become a research hotspot and are gradually being applied to the field of ICVs trajectory prediction. Cui et al. [18] proposed an autonomous driving multimodal trajectory prediction method based on deep convolutional neural networks (CNNs), which encoded the vehicle’s environment as a raster image that was input into the CNN network to output the predicted trajectory of the vehicle. Luo et al. [19] proposed a single vehicle trajectory prediction method based on CNN networks to extract the motion features of vehicles in point cloud data, and then a new convolutional layer was added to achieve the prediction of the vehicle trajectories. Since single neural networks cannot satisfy the requirements of vehicle trajectory prediction [20], many scholars focus on hybrid neural networks. When considering the temporal features of trajectories in roadway scenarios, Qin et al. [21] proposed a Q-LSTM model to reduce the collision phenomenon. Better prediction performance is obtained by optimizing the LSTM network parameters. With the widespread application of vehicle-to-vehicle (V2V) and vehicle-to-infrastructure (V2I) communication technologies [22,23,24], the accuracy of trajectory prediction under urban scenarios has been improved greatly by combining the advantages of sensor fusion technologies. Zyner et al. [25] proposed a trajectory prediction method based on multimodal probabilistic solutions, which combined recurrent neural networks (RNNs) with mixture density networks (MDNs) to predict vehicle trajectories with high prediction accuracy. Schreiber et al. [26] proposed a method to fuse RNN networks with LiDAR grids. The top view was used as the input of the RNN networks, and better predictions were achieved by optimizing the weighted parameters of the network. When considering the mobility, interaction, and similarity of the vehicles, and the problem of gradient explosion and gradient disappearance when applying long sequence data to the training process, Ma et al. [27] proposed a real-time trajectory prediction model based on long short-term memory (LSTM) to refine the predicted vehicle trajectory types. Ji et al. [28] proposed a vehicle trajectory prediction method for the forced lane changes of vehicles in a weaving area, which considered the multimodal characteristic of vehicle motion. The experimental results showed that it had higher prediction accuracy in the lane changes of autonomous vehicles for trajectory prediction when compared with the model-based traditional methods.

In summary, the existing ICVs technology applications have rich research achievements in target detection, tracking, and trajectory prediction. However, how to combine microscopic V2X driving dynamic data and multisource environment data to further improve vehicle positioning accuracy and real-time trajectory prediction capabilities under urban intersection scenarios still needs to be further studied. The key contributions of this paper are summarized as follows:

1. We designed a real-time trajectory prediction method for ICVs, which combines the advantages of the Q-Learning algorithm and LSTM network with more consideration of spatiotemporal characteristics. We utilized the GM-PHD model to fuse the multi-sensor data output from the camera, LiDAR, V2X unit and traffic signal controller. Therefore, we not only enhanced positioning capabilities but also improved the capability of the trajectory prediction of the ICV;
2. We improved the dimensionality of the input of an improved LSTM model by using microscopic data from V2X communication, such as speed, acceleration, and traffic light timing data. Meanwhile, the signal light factor was considered in the improved LSTM model, and the proposed trajectory prediction method had better performance at signal-controlled intersections;
3. Different from most previous research results on vehicle trajectory prediction, we constructed an intelligent roadside unit for perceiving the data states of the ICVs, such as latitude, longitude, altitude, acceleration, and the trajectories of the ICVs, which could be predicted. Meanwhile, a practical urban intersection was selected for testing and evaluating the performance of the proposed model, obtaining a more credible result than the simulation.

The remainder of this paper is organized as follows. In Section 2, combined with V2X communication, an MSDF model based on GM-PHD and an improved LSTM model based on Q-Learning are presented. In Section 3, the experimental results of the proposed model are demonstrated and analyzed. Finally, In Section 4, the conclusions from this paper, along with the aspects of future work, are introduced.

## 2. Real-Time Trajectory Prediction Method for Intelligent Connected Vehicles

**Figure 1:**
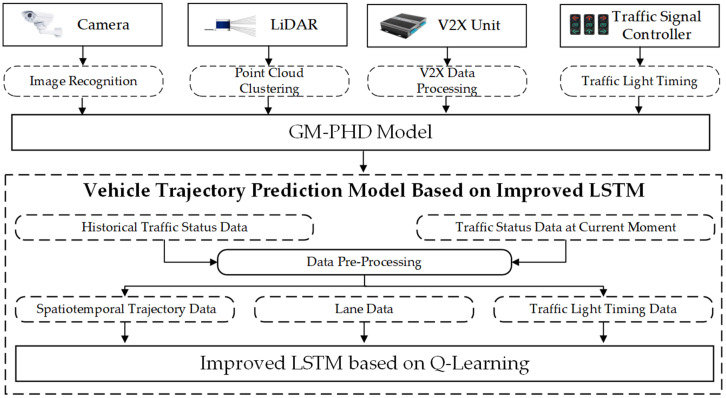
Structure of real-time trajectory prediction method.

In this section, we present a real-time trajectory prediction method based on V2X communication, which is shown in Figure 1. Multisource data were obtained by the camera, LiDAR, V2X unit, and traffic signal controller as the input of the GM-PHD model to achieve ICV perception. When combined with the historical traffic state data, which had been preprocessed, the spatial-temporal trajectory information of the ICVs was obtained via an improved LSTM model.

The proposed method includes two parts: (1) a vehicle perception model based on GM-PHD; (2) a vehicle trajectory prediction model based on improved LSTM. Based on GM-PHD theory, we collected the ICVs state data, which were applied to improving the LSTM model. Q-Learning was then added to the LSTM to realize the real-time trajectory prediction of the ICVs.

### 2.1. Vehicle Perception Model Based on GM-PHD

**Figure 2:**
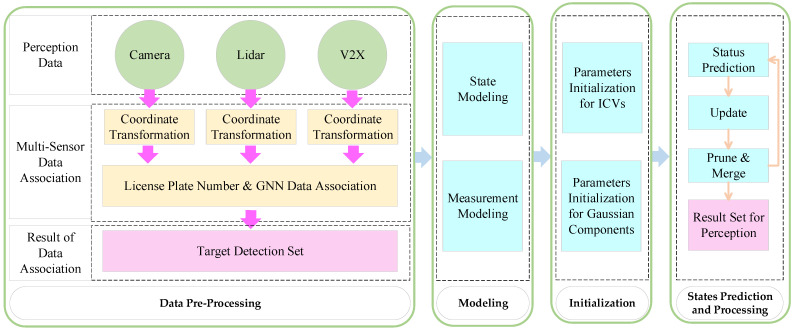
Processing of vehicle perception based on GM-PHD model.

The perception model has two parts: (1) data preprocessing and the (2) GM-PHD model. The specific processing is shown in Figure 2. GM-PHD is a multiple object tracking (MOT) model that can adapt to fuse multi-sensor data and apply this to the situation, with varying numbers of the ICVs. The processing consisted of: (1) data preprocessing; (2) modeling; (3) initialization, and (4) state prediction and processing.

(1) Data preprocessing

The image data were detected by the YOLOv5 algorithm to obtain information on the ICV states. When considering that the point cloud has a large amount of data, the VoxelGrid [29] filtering algorithm was selected to reduce the data load. Then, the target-level perception data of the multi-sensor were transformed by perspective-n-point (PNP) and camera calibration to a global co-ordinate system. The timestamps of the sensory data are aligned by linear interpolation, and the image data are matched with the V2X communication data by license plate number. In addition, the global nearest neighbor (GNN) algorithm was applied to fuse the data of the camera, LiDAR, and V2X, and the state of ICVs as output.

(2) The Modeling of ICVs

The geodetic coordinate system was selected as the reference of the ICVs, with an *x*-axis along the road direction and a *y*-axis along the vertical road direction. Measurement data [*x*, *y*, *v*_x_, *v*_y_, *a*_x_, *a*_y_, *δ*], which were acquired by V2X communication, were added to improve the accuracy of the tracking. We define $N^{\mathrm{obj}}$ as the number of ICVs at time *k* and we define ***X**_k_* as the set of ICV states $X_{k}=\{x_{1,k},x_{2,k},\ldots ,x_{i,k},\ldots ,x_{N^{obj}(k),k}\}$, where the state vector $x_{i,k}$ at time *k* constitutes of the position, velocity, and acceleration. The definition is shown in Equation (1), and the updating equation is defined in Equation (2).

(1) $$x_{i,k}={\left[\begin{array}{cccc} x & y & v_{x} & v_{y} \end{array}\right]}^{T},i\in N^{\mathrm{obj}}(k)$$

(2) $$x_{i,k+1}=F_{k}x_{i,k}+\varepsilon_{k}$$

where [*x*, *y*] indicates the vector of the vehicular position and [*v*_x_, *v*_y_] indicates the vector of the vehicular speed. ***ε**_k_* represents Gaussian white noise, which covariance follows the normal distribution *N*(**·**, ***R***), and ***F****_k_* indicates the state transition matrix.

The number of perceived ICVs at time *k* is defined as *N_s_*(*k*), then all the observed ICVs at the intersection can be represented by the measurement data set $Z_{k}=\{z_{1,k},z_{2,k},\ldots ,z_{i,k},\ldots ,z_{N_{s}(k)}\}$. The observed vector of the vehicle *i* state at time *k* is defined as ***z****_i_*_,__*k*_, which contains perturbation, as shown in Equation (3). The observation equation of the sensors is shown in Equation (4).

(3) $$z_{i,k}={\left[\begin{array}{cccc} x & y & v_{x} & v_{y} \end{array}\right]}^{T},i\in N_{s}(k)$$

(4) $$z_{i,k}=H_{k}x_{i,k}+\varsigma_{k}$$

where ***H_k_*** indicates the observation matrix of the linear system, and $\varsigma_{k}$ indicates the Gaussian white noise observed by the sensor, which follows the distribution of *N*(**·**, ***R***).

(3) Initialization of the GM-PHD parameters

The ICVs and potential ICVs are represented by Gaussian components {*w*, *m*, *P*, *ξ*, *n*}, which denote the weights, the mean states, the covariance matrix, the number of Gaussian components, and the classification based on the GM-PHD [13] algorithm.

(4) ICV states prediction and processing

The Kalman filter was applied to the GM-PHD algorithm to predict the Gaussian components, as shown in Equations (5)–(9). In the updated processing, the weights are updated by the observed ICVs state based on the current state *w* and the detection probability and martingale distance, as shown in Equation (10). Then, the Gaussian component is updated to obtain the new Gaussian component.

(5) $$v_{k-1}(x)=\overset{J_{k-1}}{\underset{i=1}{\sum }}w_{k-1}^{i}N\left(x;m_{k-1}^{i},P_{k-1}^{i}\right)$$

(6) $$\gamma_{k}(x)=\overset{J_{\gamma ,k}}{\underset{i=1}{\sum }}w_{\gamma ,k}^{i}N\left(x;m_{\gamma ,k}^{i},P_{\gamma ,k}^{i}\right)$$

(7) $$w_{k|k-1}^{i}=w_{k-1}^{i}$$

(8) $$m_{k|k-1}^{i}=F_{k-1}m_{k-1}^{i}$$

(9) $$P_{k|k-1}^{i}=Q_{k-1}+F_{k-1}P_{k-1}^{i}F_{k-1}^{T}$$

(10) $$v_{k}(x)=\left(1-P_{D,k}\right)v_{k|k-1}(x)+\underset{z\in Z_{k}}{\sum }v_{D,k}(x;z)$$

where $v_{k-1}$ indicates the intensity function of the ICVs at time *k*−1, *J_k−_*_1_ indicates the number of Gaussian components, and $N\left(x;m_{k-1}^{i},P_{k-1}^{i}\right)$ indicates the distribution of *i*-th Gaussian components. $w_{k-1}^{i}$, $m_{k-1}^{i}$, $P_{k-1}^{i}$ indicate the weights, mean, covariance matrix of the distribution of Gaussian components, ***F****_k_* indicates the matrix of state transition, $P_{\gamma ,k}^{i}$ indicates the covariance matrix described by the distribution of $v_{k-1}$ near the peak $m_{\gamma ,k}^{i}$; $w_{\gamma ,k}^{i}$ indicates the weights of the number of newborn ICVs; *P_D_*_,__*k*_ indicates the detection probability of the vehicle; $v_{D}{}_{,k}$ indicates the posterior density of the detected ICVs; $\left(1-P_{D}{}_{,k}\right)v_{k}{}_{|k-1}\left(x\right)$ indicates the intensity of the undetected ICVs; $\underset{z\in Z_{k}}{\sum }v_{D,k}(x;z)$ indicates the intensity of the detected ICVs by the sensors, and $\gamma_{k}(x)$ indicates the newborn ICVs intensity at the intersection.

Moreover, a large number of computational resources can be consumed in complex scenarios with background noise, interference, and measurements. Therefore, we cite the method introduced by Lindenmaier [30] to prune the Gaussian components, and the accurate ICVs data states at the intersection were obtained. The pseudocode of GM-PHD algorithm is shown in Table 1.

### 2.2. Vehicle Trajectory Prediction Model Based on Improved LSTM

**Figure 3:**
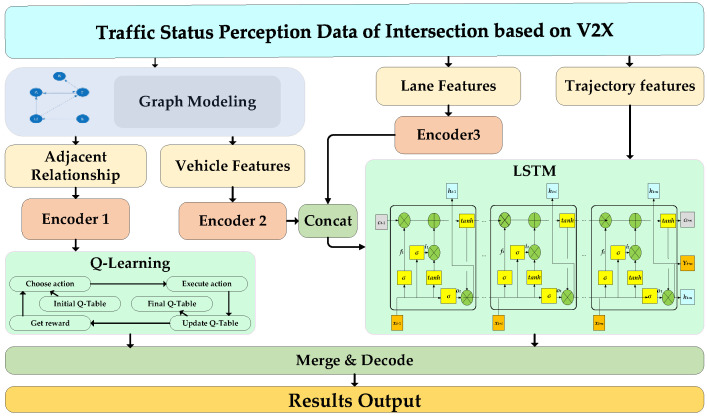
Structure of improved LSTM model for ICVs.

When combined with the states of the ICVs outputted by improved GM-PHD and signal light states, we applied graph modeling and an encoding unit before LSTM. The feature of V2X communication data, which can be acquired under the connected scenarios, is compressed to unify the feature dimensions. Then, considering the positional relationship between vehicle-to-vehicle, the Q-Learning algorithm was selected to gain the features of spatial dimension. LSTM was selected to gain the features of the temporal dimension. After the processing of merge and decoding, the trajectories of the ICVs could be predicted by the features. The structure of the improved LSTM model is shown in Figure 3.

#### 2.2.1. Graph Modeling and Features Encoding for Improved LSTM

The number of ICVs is defined as *N*, and each ICV is defined as a node from the graph. The node feature matrix ***X*** consists of position coordinates (*x*, *y*), velocity *v*, acceleration *a*, heading angle *φ*, body length *L*, body width *W*, and signal light factor *T*_L_, which is shown in Equation (11). The fixed co-ordinate system was selected to unify the co-ordinate system, and the *x*-axis direction of the ICVs is defined as the road direction, the *y*-axis is vertical to the *x*-axis, and the co-ordinate system obeys the right-handed system rule.

(11) $$X=\left[\begin{array}{c} X_{1}\\ X_{2}\\ \begin{array}{c} \vdots \\ X_{i}\\ \vdots \end{array}\\ X_{n} \end{array}\right]=\left[\begin{array}{ccccccc} x_{1} & y_{1} & v_{1} & a_{1} & \varphi_{1} & L_{1} & W_{1}\\ x_{2} & y_{2} & v_{2} & a_{2} & \varphi_{2} & L_{2} & W_{2}\\ \vdots & \vdots & \vdots & \vdots & \vdots & \vdots & \vdots \\ \begin{array}{c} x_{i}\\ \vdots \\ x_{n} \end{array} & \begin{array}{c} y_{i}\\ \vdots \\ y_{n} \end{array} & \begin{array}{c} v_{i}\\ \vdots \\ v_{n} \end{array} & \begin{array}{c} a_{i}\\ \vdots \\ a_{n} \end{array} & \begin{array}{c} \varphi_{i}\\ \vdots \\ \varphi_{n} \end{array} & \begin{array}{c} L_{i}\\ \vdots \\ L_{n} \end{array} & \begin{array}{c} W_{i}\\ \vdots \\ W_{n} \end{array} \end{array}\begin{array}{c} T_{L}{}_{1}\\ T_{L}{}_{2}\\ \vdots \\ \begin{array}{c} T_{L}{}_{i}\\ \vdots \\ T_{L}{}_{n} \end{array} \end{array}\right]$$

where *T*_L_ indicates the signal light factor, which can be introduced as the remaining time of the red light when the ICV arrives at the next crosswalk maintaining constant velocity. The parameters [*x_i_*, *y_i_*, *v_i_*, *a_i_*, *φ_i_*, *L_i_*, *W_i_*] (*i* = 1, 2, 3, …, *n*) of matrix ***X*** can be obtained from the V2X fusion perception trajectory information.

The adjacency matrix ***G*** of the graph is shown as Equations (12) and (13).

(12) $$G=\left[\begin{array}{cccc} g_{11} & g_{12} & \cdots & g_{1n}\\ g_{21} & g_{22} & \cdots & g_{2n}\\ \vdots & \vdots & \ddots & \vdots \\ g_{n1} & g_{n2} & \cdots & g_{nn} \end{array}\right]$$

(13) $$g_{ij}=\left\{\begin{array}{l} D(i,j),i\neq j\\ 0,i=j \end{array}\right.,D(i,j)=\sqrt{{\left(x_{i}-x_{j}\right)}^{2}+{\left(y_{i}-y_{j}\right)}^{2}}$$

where *g_ij_* indicates the Euclidean distance between the vehicles. The heading angle of the ICVs can be directly obtained from the V2X perception data.

Both the input and output trajectory prediction data of the ICVs are shown in Equation (14).

(14) $$\begin{array}{l} P_{r}=[(x^{t},y^{t}),(x^{t-1},y^{t-1}),\ldots ,(x^{t-\alpha_{\mathrm{in}}},y^{t-\alpha_{\mathrm{in}}})]\\ P_{f}=\Lambda (P_{r})=[(x^{t},y^{t}),(x^{t+1},y^{t+1}),\ldots ,(x^{t+\beta_{\mathrm{out}}},y^{t+\beta_{\mathrm{out}}})] \end{array}$$

where **Λ** indicates the mapping of the historical trajectory space to the prediction trajectory space, *α*_in_ indicates the number of historical trajectory points, and *β*_out_ indicates the number of predicted trajectory points at time *t*.

#### 2.2.2. Prediction of ICVs Trajectory Based on the LSTM Model

**Figure 4:**
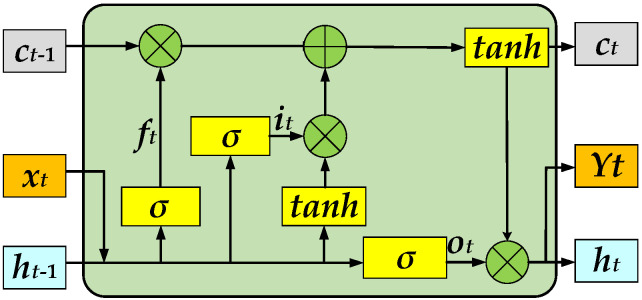
The LSTM model in the trajectory prediction of the ICVs.

In the time dimension, LSTM (with a deep structure) has the memory unit for storing historical time-series information, and the structure of the LSTM model is shown in Figure 4.

In Figure 4, the vehicle features, lane feature, and signal timing information are adopted as the input of the LSTM model. The input gates, forget gates, and output gates as the constraint control of ICVs, are provided by the model. Moreover, parts of trajectory features can be forgotten by forget gates, and the new features obtained by the Sigmoid function *σ* and hyperbolic tangent function tanh are added to the LSTM instead of the trajectory features that are discarded in the forgetting gates, as shown in Equations (15) and (16).

(15) $$\sigma (x)=\frac{1}{1+e^{-x}}$$

(16) $$\tanh(x)=\frac{2}{1+e^{-2x}}-1$$

The calculation process of LSTM is summarized as follows:

(17) $$f_{t}=\sigma (W_{\mathrm{xf}}x_{t}+W_{\mathrm{hf}}h_{t-1}+b_{f})$$

(18) $$i_{t}=\sigma (W_{\mathrm{xi}}x_{t}+W_{\mathrm{hi}}h_{t-1}+b_{i})$$

(19) $$o_{t}=\sigma (W_{\mathrm{xo}}x_{t}+W_{\mathrm{ho}}h_{t-1}+b_{o})$$

(20) $$c_{t}=f_{t}c_{t-1}+i_{t}tanh(W_{\mathrm{xc}}x_{t}+W_{\mathrm{hc}}h_{t-1}+b_{c})$$

(21) $$h_{t}=o_{t}tanh(c_{t}^{})$$

where [***W***_xc_, ***W***_xo_, ***W***_xi_, and ***W***_xf_]^T^ indicate the weight matrix of vehicle feature, [***W***_hc_, ***W***_ho_, ***W***_hi_, and ***W***_hf_]^T^ indicates the weight matrix of the hidden layer, ***x****_t_* indicates the input value of the node features of the ICVs at time *t*, [***b***_c_, ***b***_o_, ***b***_i_, ***b***_f_]^T^ indicates the offset vector and ***h****_t_*_−1_ indicates the output value of vehicle trajectory sequence at time *t*−1. In Equation (17), ***f****_t_* (***f****_t_* ∈ [0, 1]) indicates the state of the forget gate. In Equation (18), ***i****_t_* (***i****_t_* ∈ [0, 1]) indicates the state of the input gate. In Equation (19), ***o****_t_* (***o****_t_* ∈ [0, 1]) indicates the state of the output gate. In Equation (20), ***h****_t_* indicates the output of the LSTM in Equation (21).

#### 2.2.3. Improved LSTM Based on Q-Learning

**Figure 5:**
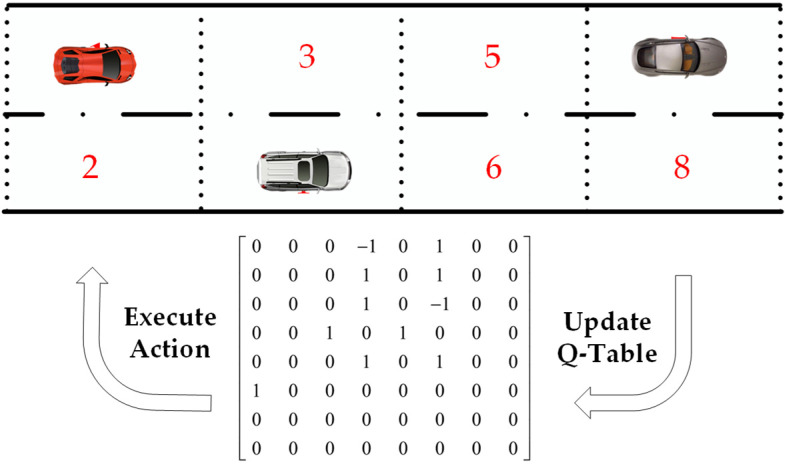
Structure of Q-Learning in ICVs trajectory prediction.

When combined with the feature of ICV spatial distribution, the Q-Learning algorithm was selected in this section to optimize the LSTM model. Q-Learning is the value-based reinforcement learning algorithm, one of the key parameters ***Q***(*s*, *m*) denotes the expectation that the benefit can be obtained by the action *m* ∈ *M*, and the corresponding reward can be the feedback, according to the action set *M* of the ICVs. The optimal route, which is stored in the ***Q***-table, can be selected to obtain the maximum benefit action. The structure of Q-Learning is shown in Figure 5.

The Q-Learning algorithm can be integrated with the LSTM model for the purpose of accurate predicting the ICV trajectory. Meanwhile, the road is coded in a grid pattern, and each of the road grids is defined as a road node with red node numbers in Figure 5. The processing of the algorithm is shown as follows:

Step 1: Initialize the action value function ***Q***(*s*, *m*);

Step 2: A new action *m* is selected by the ICV, according to the Q-greedyUCB policy [31] and executing;

Step 3: Reward *r* is received by the ICV, and a new state *s* + 1 is selected;

Step 4: Updating the $Q^{*}(s,m)$ function;

Step 5: Repeating Steps 2–4 until the ICV reaches the expectation states of ICV;

Step 6: Output the last generated path scheme of the ICV.

The updated of $Q^{*}(s,m)$ function is shown as Equation (22).

(22) $$Q^{*}(s,m)=(1-\mu )Q(s,m)+\mu \left(r+\gamma \underset{m+1\in M}{max}Q(s+1,m+1)\right)$$

where *μ* (*μ* ∈ [0, 1]) indicates the learning rate of the Q-Learning algorithm, *γ* (*γ* ∈ [0, 1]) indicates the discount factor, which can make the algorithm pay more attention to the current or future reward, ***Q***(*s*, *m*) indicates the current reward under the current state for the current action, $Q^{*}(s,m)$ indicates the desired maximum reward obtained by the ICVs.

**Figure 6:**
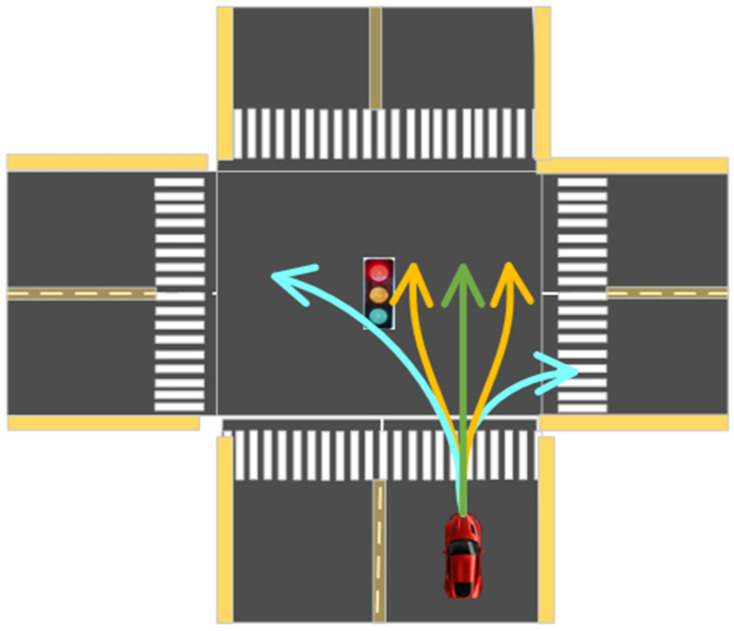
Diagram of ICV action, including straight ahead, left lane change, right lane change, left turn, and right turn.

Generally, the ICVs may have five actions (straight ahead, left lane change, right lane change, left turn, and right turn) at an intersection, as shown in Figure 6.

After action *m* is executed, if the ICV cannot reach the target grid, the Q-value is set as 0. Otherwise, the Q-value configurations are shown in Table 2. In addition, The ***Q***-table of the ICVs at initial time *t* is shown in Equation (23).

(23) $$Q=\left[\begin{array}{cccccccc} 0 & 0 & 0 & 0 & 0 & 0 & \cdots & 0\\ 0 & 0 & 0 & -1 & 1 & 0 & \cdots & 0\\ 0 & 0 & 0 & 0 & 0 & 0 & \cdots & 0\\ 0 & 0 & 0 & 0 & 0 & 0 & \cdots & 0\\ 0 & 0 & 0 & 0 & 0 & 0 & \cdots & 0\\ 0 & 0 & 0 & 0 & 0 & 0 & \cdots & 0\\ \vdots & \vdots & \vdots & \vdots & \vdots & \vdots & \ddots & \vdots \\ 0 & 0 & 0 & 0 & 0 & 0 & \cdots & 0 \end{array}\right]$$

**Figure 7:**
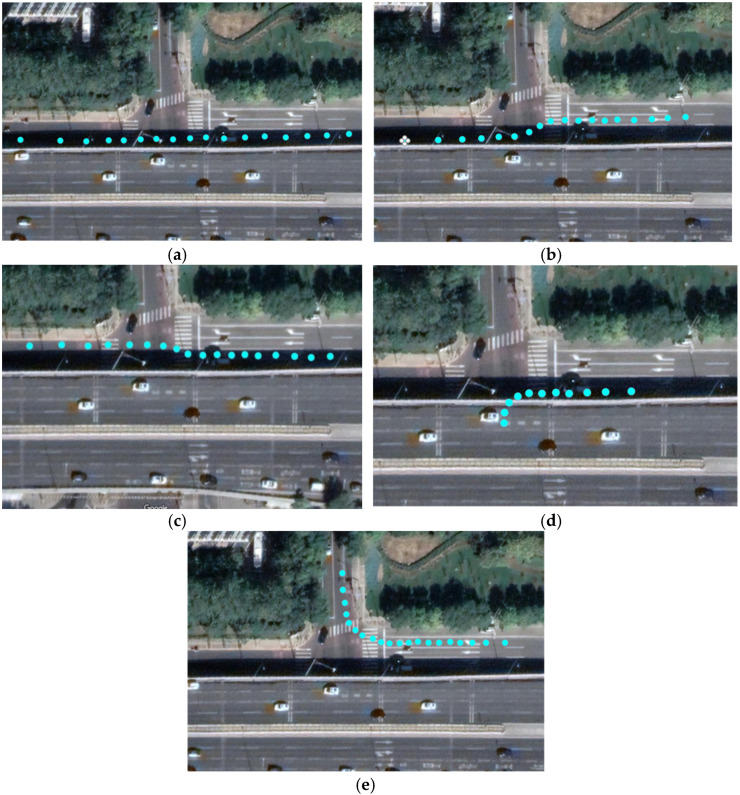
Driving behaviors and trajectories of ICVs at the intersection. (**a**) straight ahead; (**b**) left lane change; (**c**) right lane change; (**d**) left turn; (**e**) right turn.

The route with less time cost is defined as a better scheme for ICVs. In this section, the Q-greedyUCB algorithm [31] is selected as the action policy in the Q-Learning algorithm. In the processing of LSTM model training, five driving behaviors (straight ahead, left lane change, right lane change, left turn, and right turn) are considered to achieve trajectory prediction. The trajectories of the ICVs at the intersection are shown in Figure 7.

The weight matrix and offset vector of the vehicle features are obtained by training the LSTM model, and the loss function of the LSTM is shown in Equation (24).

(24) $$J_{1}=-\overset{T}{\underset{t=1}{\sum }}\ln P_{\tau }\left(\upsilon^{\left(t\right)}|\upsilon_{h}\right)$$

where υ(t) indicates the predicted trajectory at time *t*, and *τ* indicates the parameters of the weight matrix and offset vector in the LSTM model. $\upsilon_{h}$ indicates the vector of historical trajectory feature.

The trajectory prediction by LSTM needs to be optimized in combination with the Q-Learning algorithm. The loss function of Q-Learning combined with the LSTM is designed to fuse the vehicle trajectory behavior features and the driving features of the ICV, as shown in Equation (25).

(25) $$\begin{array}{ll} J_{2} & =\overset{T}{\underset{t=1}{\sum }}D_{\alpha }\left(P^{\mathrm{lstm}}\left(\upsilon^{\left(t\right)}\right)∥Q^{\mathrm{ql}}\left(\upsilon^{\left(t\right)}\right)\right)\\ & =\overset{T}{\underset{t=1}{\sum }}\frac{4}{1-\alpha^{2}}\left(1-\int P^{\mathrm{lstm}}{\left(\upsilon^{\left(t\right)}\right)}^{\frac{1+\alpha }{2}}Q^{ql}{\left(\upsilon^{\left(t\right)}\right)}^{\frac{1-\alpha }{2}}d\upsilon^{\left(t\right)}\right) \end{array}$$

where *P*^lstm^ indicates the probability functions of the predicted trajectories in LSTM, *Q*^ql^ indicates the probability functions of the predicted trajectories in Q-Learning, and *D_α_* indicates the *α* divergence. When considering the symmetry of *D_α_*, we set *α* as 0 to make the LSTM and Q-Learning prediction results as similar as possible. Finally, the loss function can be defined as Equation (26) by combining *J*_1_ and *J*_2_.

(26) $$J=\beta J_{1}+(1-\beta )J_{2}$$

where *β* (*β* ∈ [0, 1]) indicates the ratio of *J*_2_ in the final loss function.

## 3. Results and Discussion

In this section, a field test scenario for the ICVs was constructed based on an intelligent roadside unit, and the parameters of the model and scenario are listed in detail. Then, the evaluation metrics of GM-PHD and the improved LSTM model are introduced to verify and analyze the advanced of proposed model.

### 3.1. Scenario and Parameters

**Figure 8:**
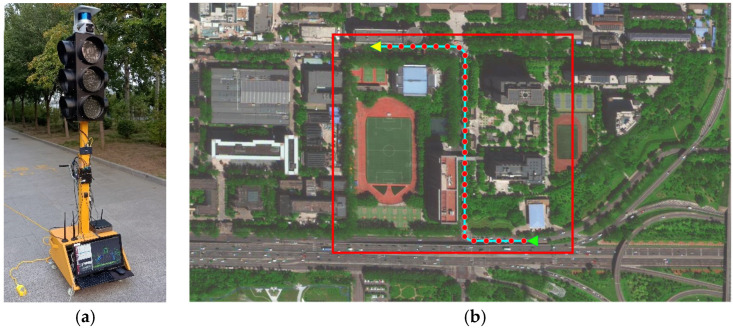
Experimental scenarios and equipment. (**a**) roadside unit; (**b**) experimental scenarios.

The ICVs and road infrastructure have real-time data-exchange capabilities via the V2X unit. DSMP (LTE-V communication protocol) was adopted by the roadside unit (RSU) to communicate with the on-board unit (OBU). Meanwhile, an intersection on the auxiliary road of Fushi Road in Shijingshan District, Beijing, was selected as the experimental scenario. The time of the experiments was selected between 7:00 and 19:30, and the saturation flow of the intersection was 319.04 pcu/h. The top view of the experimental scenarios is shown in Figure 8b.

According to the survey of the selected scenarios, the experimental scenarios occupied 350 × 350 m^2^ areas, which is marked in red rectangle block in Figure 8b, and the driving route of the ICVs is shown as an example. The driving route includes three types of driving behaviors: straight ahead, right turn, and left turn, where the green “△” indicates the origin point of the vehicle and the yellow “△” indicates the destination point of the driving. In addition, in order to verify the detection accuracy, we adopted the centimeter-level positioning data of the ICVs as the ground truth value.

Moreover, an intelligent roadside unit was deployed beside the intersection, which is equipped with a gigabit switch, cameras, LiDAR, V2X units, and mobile edge computing (MEC). A high-performance embedded processor with 30 Tops as the MEC device could ensure the speed and efficiency of algorithm execution. The intelligent roadside unit is shown in Figure 8a. The list of configurations is shown in Table 3.

### 3.2. Evaluation Metrics

The performance of trajectory prediction is susceptible to the perceptional accuracy of the ICVs, and to evaluate the perceptional accuracy, the mean absolute percentage error (MAPE), the mean absolute error (MAE), and root mean square error (RMSE) are used in this paper, as shown in Equations (27)–(29).

(27) $$MAPE=\frac{100\%}{n}\overset{n}{\underset{i=1}{\sum }}\left|\frac{{\hat{y}}_{i}-y_{i}}{y_{i}}\right|$$

(28) $$MAE=\frac{1}{q}\overset{q}{\underset{i=1}{\sum }}\left|h_{i}-l_{i}\right|$$

(29) $$RMSE=\sqrt{\frac{1}{m}\overset{m}{\underset{i=1}{\sum }}{\left({\hat{y}}_{i}-y_{i}\right)}^{2}}$$

where ${\hat{y}}_{i}$ indicates the output of fusion positioning, $y_{i}$ indicates the actual position of the vehicle, *m* indicates the sample numbers of MAPE, *q* indicates the number of points, ***h****_i_* indicates the *i*-th point of predicted trajectory, and ***l****_i_* indicates the ground truth of the *i*-th point of trajectory.

For the evaluation of the proposed prediction model, the average displacement error (ADE) and final displacement error (FDE) are adopted as the evaluation metrics. The ADE is the average Euclidean distance between the predicted trajectory and the real trajectory. FDE is defined as the Euclidean distance between the end-point of the predicted trajectory and the end-point of the actual trajectory. The ADE and FDE functions are shown in Equations (30) and (31).

(30) $$ADE=\sqrt{\frac{\sum_{k=1}^{r}\sum_{i}^{n}\;{\left(D_{i}{}^{dist}\right)}^{\left(k\right)}}{n}}$$

(31) $$FDE=\sqrt{{\left(x^{\mathrm{pred}}-x^{\mathrm{truth}}\right)}^{2}+{\left(y^{\mathrm{pred}}-y^{\mathrm{truth}}\right)}^{2}}$$

where *n* indicates the number of vehicles, *r* indicates the prediction step, *D_i_*^dist^ indicates the Euclidean distance between the actual and predicted coordinates of vehicle *i*, [*x*^pred^, *y*^pred^]^T^ indicates the end point of the predicted trajectory, and [*x*^truth^, *y*^truth^]^T^ indicates the end point of the actual trajectory.

### 3.3. Experimental Results and Analysis

There are three experimental ICVs, with a maximum speed of 35 km/h. The three ICVs track the route in Figure 8. The range of V2X communication between the RSU and the ICVs is considered to be 300 m. The real-time traffic states, including the ICV dataset and the signal light state dataset, can be perceived by intelligent roadside unit. There are 12,403 data states for the vehicles and 1151 data states for the signal light in the dataset; parts of the dataset are shown in Table 4 and Table 5.

#### 3.3.1. Accuracy of ICV Perception Analysis

**Figure 9:**
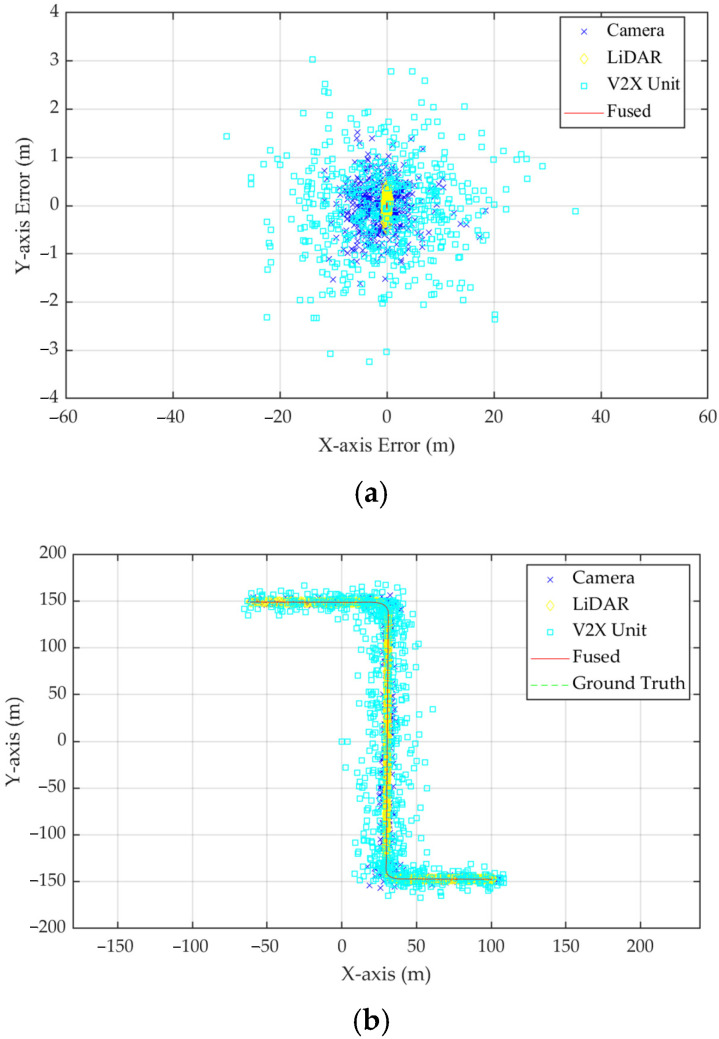
The error distribution and detection results of ICV perception. (**a**) the error distribution of the single-sensor model; (**b**) the detection results of the single-sensor model and the GM-PHD model.

A tested route was set for the ICVs, which is described in Figure 8b, and consequently, a series of perception data were recorded. The ICV perception results were perceived by the camera, LiDAR, V2X unit, the GM-PHD model, and the ground truth position, and the errors were selected to mark the map, which is shown in Figure 9.

The error distribution of the single-sensor model presents an irregular elliptical distribution, and it is more dispersed compared with the error distribution of the fused model. Thus, the vehicular detection information after fusion processing is closer to the real results, and the statistic of perception error is shown in Table 6.

The perceptional accuracy of the ICVs applying the GM-PHD model is more advantageous when compared with the single sensor. The maximum, minimum, and average error of the LiDAR has better performance when compared with the camera and V2X unit. When compared to LiDAR, the minimum error of the GM-PHD model is reduced by 86.58%. Moreover, the average error of the GM-PHD model is 0.1181 m, which is a 44.05% reduction compared to the LiDAR. By combining the data from multiple sources, the data noise is reduced, the outliers are eliminated, and the biases of each individual sensor data are corrected. Thus, when compared with the perception result of the single sensors, the perception results can be described more accurately by the fusion of the data from different sensors. Finally, the accuracy analysis of perception can further verify that the data obtained by GM-PHD has enough credibility.

**Figure 10:**
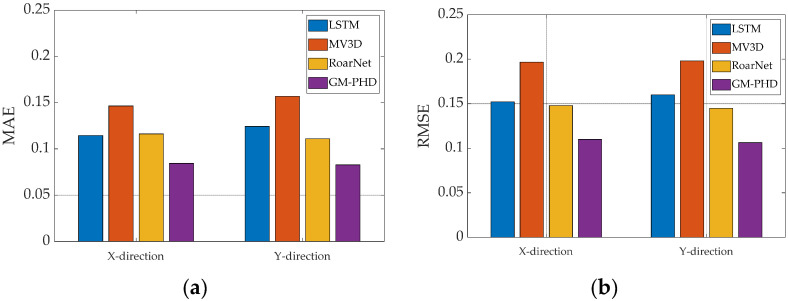
Comparison of MAE and RMSE between the LSTM, MV3D, RoarNet, and GM-PHD models. (**a**) the comparison results under MAE metric; (**b**) the comparison results under RMSE metric.

In order to evaluate the performance of the GM-PHD model, we compared the GM-PHD model with the LSTM model [32], MV3D (Multi-View 3D) model [33], and RoarNet model [34]. The RMSE and MAE metrics were selected to evaluate the performance of the models. The comparison results are shown in Figure 10.

In Figure 10, the MAE of the GM-PHD model is 0.0843 m in the X directions, and the MAE of the GM-PHD model is 0.0828 m in the Y direction. The RMSE of the GM-PHD model is 0.1100 m in the X direction, and the RMSE of the GM-PHD model is 0.1063 m in the Y direction. When compared with the LSTM model, the MV3D model, RoarNet model, and the MAE of the GM-PHD model were reduced by 27.74%, 43.49%, and 28.77%, respectively. When compared with the LSTM model, the MV3D model, RoarNet model, and the RMSE of the GM-PHD model were reduced by 30.08%, 45.93%, and 28.18%, respectively. Therefore, when compared with the LSTM model, the MV3D model, RoarNet model, and the GM-PHD model have better performance with lower MAE and RMSE values.

#### 3.3.2. Advanced ICV Trajectory-Prediction Analysis

In order to evaluate the performance of the improved LSTM model for real-time trajectory prediction, the RNN encoder-decoder (RNN ED) model [35], the social LSTM [36], and social attention [37] method were selected for comparisons with the proposed model. In addition, this section analyzes the stability of the proposed model in different time periods with different traffic flows and analyzes the time latency in the trajectory prediction.

**Figure 11:**
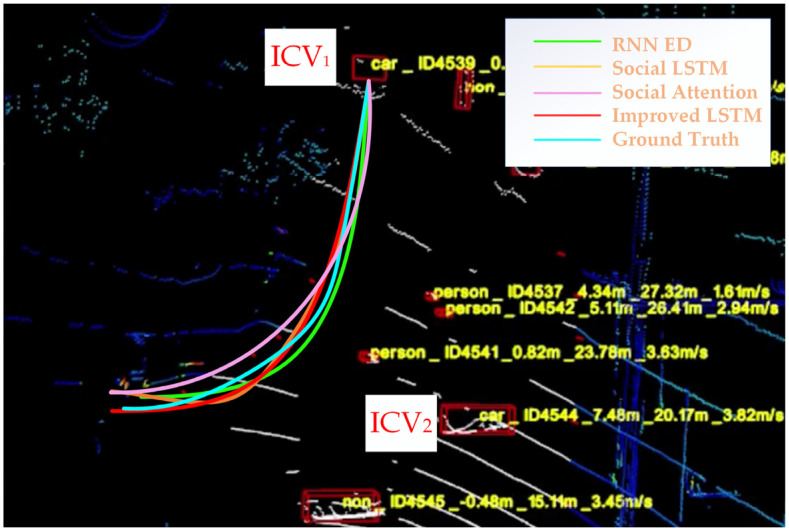
Real-time trajectory prediction results for the driving behavior of a right turn.

We deployed the intelligence roadside unit in the auxiliary road of Fushi Road, and the RNN ED, social LSTM, and social attention methods were adopted to predict the trajectory of the ICVs in the condition of driving behaviors (straight ahead, right turn, and left turn). Part of the trajectory prediction results (e.g., right turn) is shown in Figure 11.

**Figure 12:**
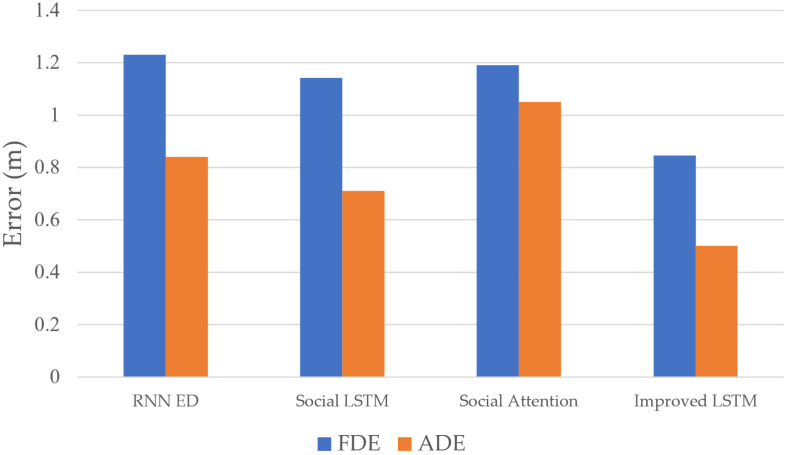
Performance of the improved LSTM model under FDE and ADE evaluation metrics.

In Figure 11, the trajectories of the ICVs that were predicted by the proposed model are shown as bold red lines, and the ground truth of the vehicle trajectories is shown as blue lines. When compared with the trajectory real-time prediction results of the RNN ED model, the social LSTM model, and the social attention model, the proposed model is closer to the actual driving trajectory of the ICV. The trajectory prediction results are statistically significant through repeated experiments. Under the FDE and ADE evaluation metrics, the results are shown in Figure 12.

In Figure 12, the error of the improved LSTM model under the FDE and ADE metrics is 0.845 m and 0.501 m, and the prediction error of the social LSTM under the ADE metric is 0.710 m. Therefore, when compared to the social LSTM model, the ADE of proposed model was reduced by 29.43%. Meanwhile, when compared with the social attention and social LSTM models, the prediction error of the improved LSTM model is smaller because the proposed model utilizes the intersection environment features, vehicle features, and V2X communication data. In summary, the proposed model can predict the ICV trajectory more accurately.

**Figure 13:**
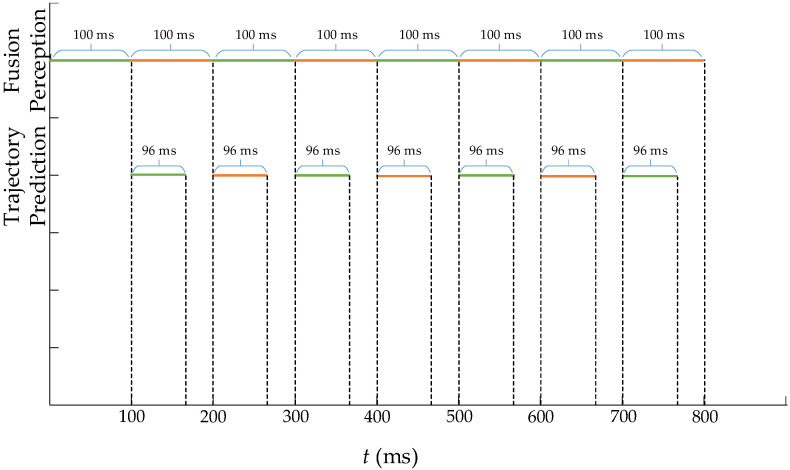
Analysis of the calculation latency of the improved LSTM model.

The system latency has an impact on the real-time performance of the system and the safety of ICVs at the intersection. In order to analyze the latency of the prediction model, the calculation latency of the improved LSTM model is shown in Figure 13.

In Figure 13, the time interval of the fusion perception is 100 ms in the system prediction processing, and the trajectory prediction model needs 96 ms of processing time to predict the trajectory of the ICVs. The total latency for the perception and trajectory prediction needs 196 ms, which is marked as same color in adjacent time period. In the processing of fusion perception, a higher number of Gaussian components need to be calculated by the prune operation of GM-PHD, leading to a reduction in work efficiency. In the trajectory prediction processing, the parameter calculations and graph modeling parts of the improved LSTM model take a certain amount of time to increase the latency of the model. When applying pipeline technology, the computation processing of the trajectory prediction is carried out simultaneously with the next computation processing of the fused perception algorithm. Thus, when considering that the time of trajectory prediction 2 s is larger than a latency of 0.196 s, the total latency satisfies the requirement of real-time trajectory prediction.

**Figure 14:**
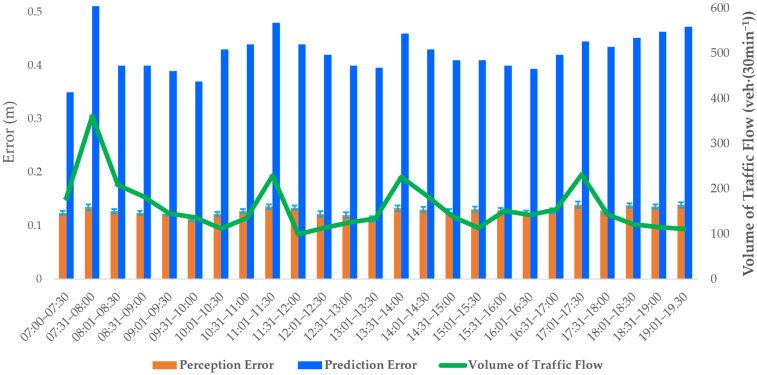
Perception and prediction errors under different traffic flows.

In order to verify the effect of traffic flow on trajectory tracking and prediction over different time periods, three ICVs were continuously tested in the intersection scenarios. The errors of prediction of the ICVs at different volumes of traffic flow in different time periods are shown in Figure 14.

In Figure 14, the orange column of the histogram indicates the perception error, and the upper and lower edges indicate the maximum and minimum errors of detection. The blue color indicates the trajectory prediction error, and the green color indicates the traffic flow volume. By analyzing the data in Figure 14, the decrease in the accuracy of prediction is due to the increase in traffic flow. However, the average displacement error of trajectory prediction is still lower than 0.501 m, so the prediction model satisfies the requirements of high-precision trajectory prediction (of ICVs). In addition, the proposed method can be extended to other similar systems, such as high-speed highway monitoring systems and tunnel monitoring systems, et al., to monitor the collision risk of vehicles on highways and ensure vehicular safety in tunnels.

## 4. Conclusions

In this paper, we focused on the intelligent perception at an urban intersection and proposed a real-time vehicular trajectory prediction method based on V2X communication; the above method was applied to an urban intersection to further improve ICV real-time trajectory and real-time prediction capabilities. When combined with V2X data, we improved the LSTM model based on the Q-Learning algorithm; the vehicle trajectory behavior features and the ICVs driving features were fused to optimize the loss function. The experimental results demonstrated that the improved LSTM model achieved an average prediction error of 0.501 m, and the error was reduced by 29.43% when compared to the social LSTM model under ADE metrics and 26.03% under FDE, which could achieve the stable and real-time prediction of ICV trajectory at different time periods and under different traffic volume flows.

In the future, in order to construct a multidimensional dataset of an intersection scenario, real-time and accurate trajectories can be provided by our achievements. Meanwhile, a more complex model for trajectory prediction will be designed and applied in challenging scenarios, such as highways, tunnels, off-ramps, and roundabouts, to improve vehicular safety and urban traffic efficiency.

## Figures and Tables

**Table 1:** Pseudocode for the GM-PHD.

1:	**Given** ${\left\{w_{{}_{i,k-1}}^{{}^{\left(v\right)}},m_{{}_{i,k-1}}^{{}^{\left(v\right)}},P_{{}_{i,k-1}}^{{}^{\left(v\right)}}\right\}}_{v=1}^{V_{i,k-1}}\;$ for target i∈{1,…,N}, the set of measurements $Z_{j,k}$ for j∈{1,…,N}
2:	**Step 1.** (Initialization)
3:	**for** i=1,…,N**do**
4:	Initialize ${\left\{w_{{}_{i,k-1}}^{{}^{\left(v\right)}},m_{{}_{i,k-1}}^{{}^{\left(v\right)}},P_{{}_{i,k-1}}^{{}^{\left(v\right)}}\right\}}_{v=1}^{V_{i,k-1}}$, Initialize $Z_{i,k}$
5:	**end for**
6:	**Step 2.** (Prediction for birth ICVs)
7:	*i*: = 0
8:	**for** $j=1,\ldots ,J_{\gamma ,k}$ **do**
9:	*i*: = *i*+1, $w_{k|k-1}^{\left(i\right)}=w_{\gamma ,k}^{\left(j\right)},m_{k|k-1}^{\left(i\right)}=m_{\gamma ,k}^{\left(j\right)},P_{k|k-1}^{\left(i\right)}=P_{\gamma ,k}^{\left(j\right)}$
10:	**end for**
11:	**for** $j=1,\ldots ,J_{\beta ,k}$ **do**
12:	**for** $q=1,\ldots ,J_{k-1}$ **do**
13:	*i*: = *i*+1, $w_{k|k-1}^{\left(i\right)}=w_{k-1}^{\left(q\right)}w_{\beta ,k}^{j}$, $w_{k|k-1}^{\left(i\right)}=d_{\beta ,k-1}^{\left(q\right)}+F_{\beta ,k-1}^{\left(j\right)}m_{k-1}^{\left(q\right)}$
14:	$P_{k|k-1}^{\left(i\right)}=Q_{\beta ,k-1}^{\left(j\right)}+F_{\beta ,k-1}^{\left(j\right)}P_{k-1}^{\left(q\right)}{\left(F_{\beta ,k-1}^{\left(j\right)}\right)}^{T}$
15:	**end for**
16:	**end for**
17:	**Step 3.** (Prediction for existing ICVs)
18:	**for** $j=1,\ldots ,J_{k-1}$ **do**
19:	*i*: = *i*+1, $w_{k|k-1}^{\left(i\right)}=p_{S,k}w_{k-1}^{\left(j\right)}$, $m_{k|k-1}^{\left(i\right)}=F_{k-1}m_{k-1}^{\left(j\right)}$, $P_{k|k-1}^{\left(i\right)}=Q_{k-1}+F_{k-1}P_{k-1}^{\left(j\right)}F_{k-1}^{T}$
20:	**end for**
21:	$J_{k|k-1}=i$
22:	**Step 4.** (Construction of PHD update components)
23:	**for** $j=1,\ldots ,J_{k|k-1}$ **do**
24:	$\eta_{k|k-1}^{\left(j\right)}=H_{k}m_{k|k-1}^{\left(j\right)}$, $S_{k}^{\left(j\right)}=R_{k}^{\left(j\right)}+H_{k}P_{k|k-1}^{\left(j\right)}H_{k}^{T}$
25:	$K_{k}^{\left(j\right)}=P_{k|k-1}^{\left(j\right)}H_{k}^{T}{\left[S_{k}^{\left(j\right)}\right]}^{-1}$, $P_{k|k}^{\left(j\right)}=\left[I-K_{k}^{\left(j\right)}H_{k}\right]P_{k|k-1}^{\left(j\right)}$
26:	**end for**
27:	**Step 5.** (Update)
28:	**for** $j=1,\ldots ,J_{k|k-1}$ **do**
29:	$w_{k}^{\left(j\right)}=(1-p_{D,k})w_{{}_{k|k-1}}^{\left(j\right)}$, $m_{k}^{\left(j\right)}=m_{k|k-1}^{\left(j\right)}$, $P_{k}^{\left(j\right)}=P_{k|k-1}^{\left(j\right)}$
30:	**end for**
31:	*l*: = 0
32:	**for** each $z\in Z_{k}$ **do**
33:	*l*: = *l*+1
34:	**for** $j=1,\ldots ,J_{k|k-1}$ **do**
35:	$w_{k}^{lJ_{k|k-1}+j}=p_{D,k}w_{k|k-1}^{\left(j\right)}N(z;\eta_{k|k-1}^{\left(j\right)},S_{k}^{\left(j\right)})$, $m_{k}^{lJ_{k|k-1}+j}=m_{k|k-1}^{\left(j\right)}+K_{k}^{\left(j\right)}(z-\eta_{k|k-1}^{\left(j\right)})$
36:	$P_{k}^{lJ_{k|k-1}+j}=P_{k|k}^{\left(j\right)}$
37:	**end for**
38:	**for** $j=1,\ldots ,J_{k|k-1}$ **do**
39:	$w_{k}^{lJ_{k|k-1}+j}:=\frac{w_{k}^{lJ_{k|k-1}+j}}{\kappa_{k}(z)+\sum_{i=1}^{J_{k|k-1}}w_{k}^{\left(lJ_{k|k-1}+i\right)}}$
40:	**end for**
41:	**end for**
42:	$J_{k}=lJ_{k|k-1}+J_{k|k-1}$
43:	**Output** ${\left\{w_{k}^{\left(i\right)},m_{k}^{\left(i\right)},P_{k}^{\left(i\right)}\right\}}_{i=1}^{J_{i,k}}$

**Table 2:** The configurations list of Q-values according to ICV states.

The Speed States of ICVs	Q-Value
Acceleration	2
Constant	1
Deceleration	−1

**Table 3:** List of configurations.

Parameters	Description	Values
Intelligent roadside unit and ICVs	The number of ICVs	3
	V2X communication	YES
	Average latency of V2X communication	6.3 ms
Sensors	Camera	1080 p/25 Hz
	LiDAR	32 lines/10 Hz
	V2X unit	LTE-V/10 Hz
GM-PHD	The updating period of the transformation equation	0.1 s
	State transition matrix of ICV $F_{k}$	$\left[\begin{array}{cccc} 1 & 0 & 0.1 & 0\\ 0 & 1 & 0 & 0.1\\ 0 & 0 & 1 & 0\\ 0 & 0 & 0 & 1 \end{array}\right]$
Improved LSTM	Number of hidden layers	3
	Number of hidden layer nodes	300
	Epoch	20
	Batch size	100
	Loss function weight *β*	0.5
	Learning rate	0.001
	Optimizer	Adam
	The number of historical trajectory points *α*_in_	30
	The number of predicted trajectories points *β*_out_	20

**Table 4:** Data states of vehicles at the intersection.

ID	Timestamp	V2X	Longitude	Latitude	Steering Angle (°)	Speed (m/s)	Acceleration (m/s^2^)	Horizontal Distance (m)	Heading Angle (°)
56	1609232645.1	Yes	116.2138744	39.9306601	2.3	0.10	−0.06	7.82	87.22
57	1609232645.1	No	116.2139378	39.9306706	——	2.12	——	12.14	89.92
⋮	⋮	⋮	⋮	⋮	⋮	⋮	⋮	⋮	⋮
66	1609233146.8	No	116.2127605	39.9306347	——	2.12	——	18.15	155.52
67	1609233146.8	No	116.2120121	39.9306501	——	4.98	——	15.47	88.59

**Table 5:** Signal light data states at the intersection.

Timestamp	Period (s)	Signal Light State (East-West)	Time Remaining (s)
1609232622	105	Green	23
1609232623	105	Green	22
⋮	⋮	⋮	⋮
1609233152	105	Red	17
1609233153	105	Red	16

**Table 6:** Performance of the GM-PHD model and the single-sensor model under maximum error, minimum error, average error, and MAPE evaluation metrics.

Evaluation Metrics	Camera	LiDAR	V2X Unit	GM-PHD
Maximum Error (m)	11.4623	0.8268	10.9980	0.1401
Minimum Error (m)	0.1917	0.0082	0.0488	0.0011
Average Error (m)	3.5881	0.2111	8.1386	0.1181
MAPE	20.26%	0.91%	28.87%	0.10%

## Data Availability

All data and models used during the study appear in this article.
